# A case of high-grade differentiated liposarcoma in the oesophagus

**DOI:** 10.1093/bjrcr/uaaf068

**Published:** 2025-12-26

**Authors:** Hongyang Li, Hui Huang, Ting Zhao, Zhuoya Ma, Licui Zhang, Yong Wen, Qinghong Duan

**Affiliations:** Medical Imaging College of Guizhou Medical University, Guiyang, Guizhou 550000, China; Medical Imaging College of Guizhou Medical University, Guiyang, Guizhou 550000, China; Medical Imaging College of Guizhou Medical University, Guiyang, Guizhou 550000, China; Medical Imaging College of Guizhou Medical University, Guiyang, Guizhou 550000, China; Medical Imaging College of Guizhou Medical University, Guiyang, Guizhou 550000, China; Medical Imaging College of Guizhou Medical University, Guiyang, Guizhou 550000, China; Medical Imaging College of Guizhou Medical University, Guiyang, Guizhou 550004, China

**Keywords:** Adiposarcoma, Oesophagus, high-grade differentiated liposarcoma

## Abstract

Liposarcoma is a rare malignancy that is typically amenable to surgical resection if there are no distant metastases.^1^ However, it has a high degree of misdiagnosis. The primary differential diagnoses are sarcoma, gastrointestinal stromal tumour (GIST), and fibrovascular polyp.^2^ The diagnosis and management of liposarcoma remain challenging due to its lack of specific clinical symptoms and the absence of standardized and effective treatment protocols. Here, we present a case of high-grade, well-differentiated liposarcoma of the oesophagus. It is hoped that this report will increase awareness of the imaging features of this rare neoplasm among radiologists and clinicians, thereby reducing the likelihood of missed diagnoses.

## Background

**Figure 1.**
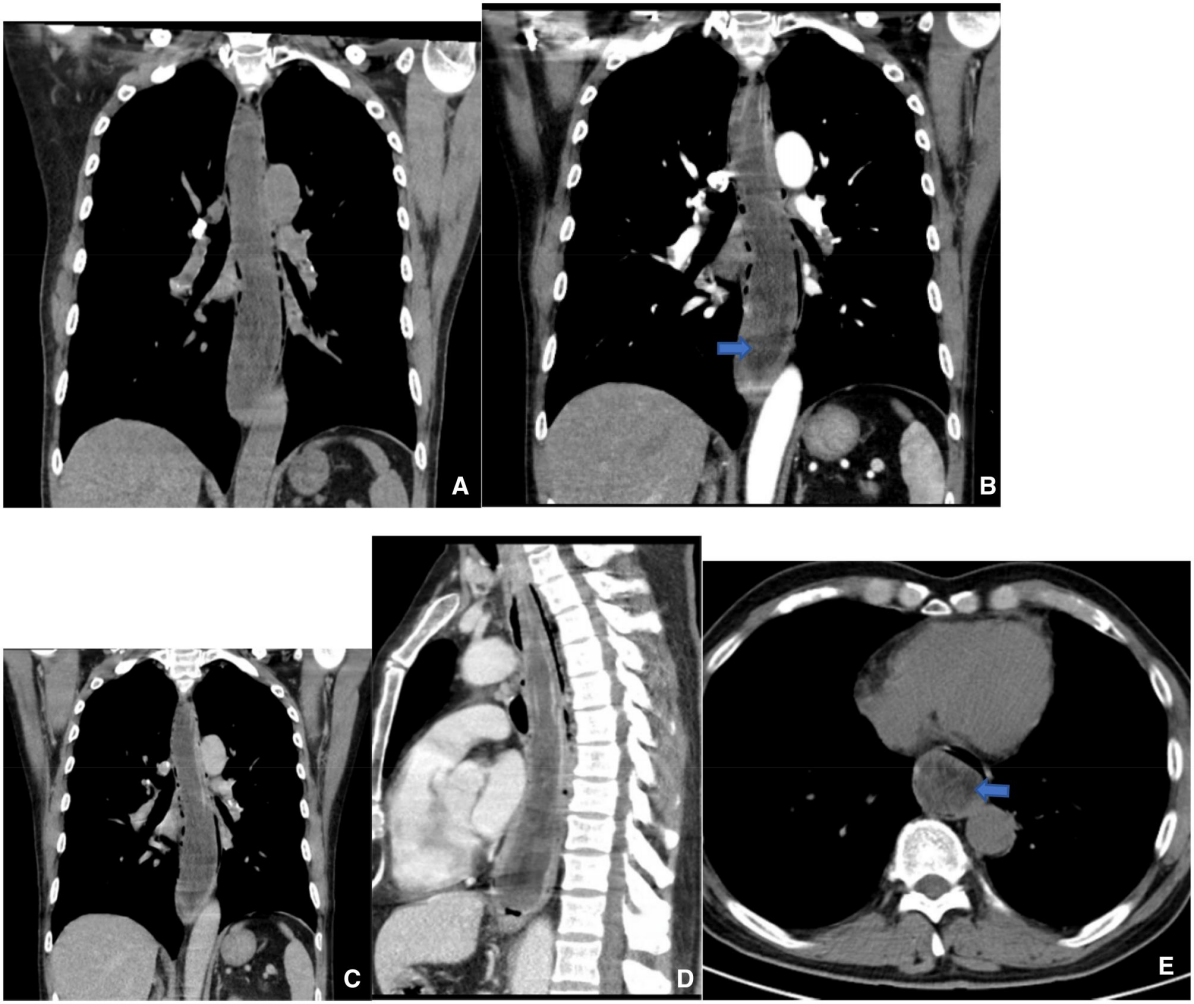
Male, 62 years old, liposarcoma. CT coronal (A, B, C) and sagittal (D) scans show a large mass in the thoracic esophagus (arrowheads), 22 mm long, with a small amount of fat within the lesion (blue arrowheads); CT coronal enhancement scan showing mild enhancement of the solid component.

A 62-year-old male was admitted with a complaint of dysphagia that had persisted for over a month and had worsened during the preceding week. The dysphagia had a progressive onset with no apparent precipitating factors and was not accompanied by chest pain, chest tightness, or dyspnea. The patient had received no treatment, and his symptoms deteriorated progressively until he experienced difficulty in swallowing food. Physical examination revealed no abnormalities. Chest computed tomography (CT) indicated an intraluminal mass within the thoracic oesophagus (Figure 1). Upper gastrointestinal endoscopy revealed a large lesion protruding into the lumen of the oesophagus. Esophagoscopy identified a massive submucosal mass extending downwards from the left oesophageal inlet to the left posterior wall, located approximately 38 cm from the incisors. The lesion caused significant narrowing of the oesophageal lumen. Narrow-band imaging (NBI) showed a type Iib mucosal pattern with teal discoloration and dilated vasculature.

**Figure 2.**
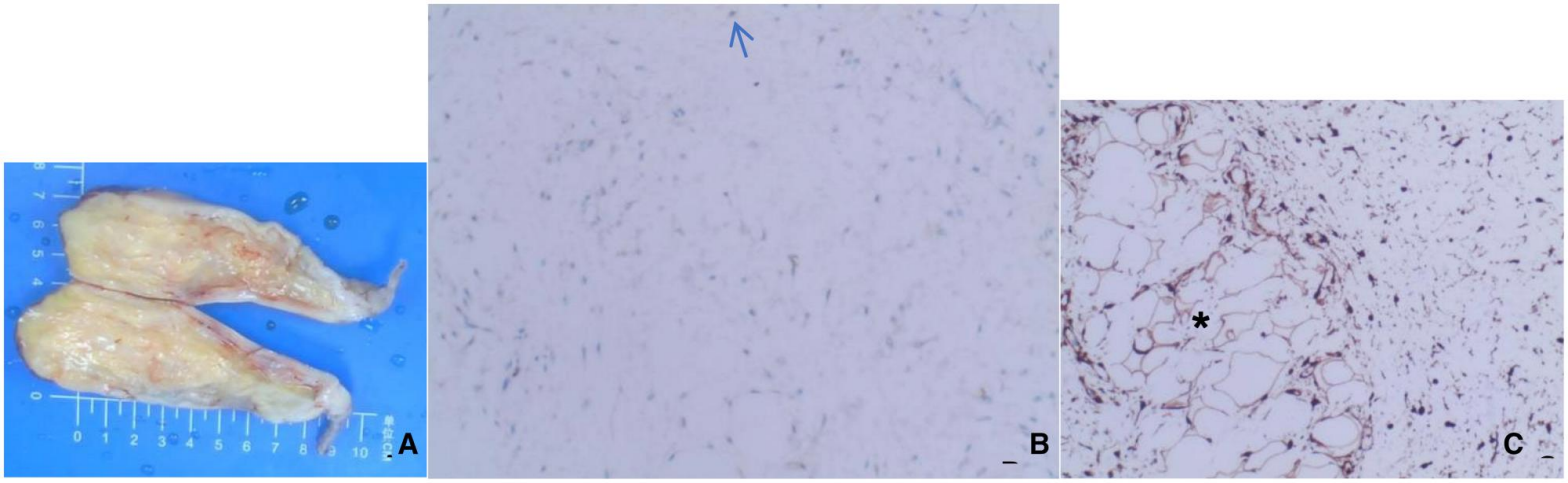
Male, 62 years old, liposarcoma. Macroscopic view of the specimen (A) shows the presence of adipose tissue, corresponding to the adipocytes shown in (C). (B) Immunohistochemical image, MDM2 immunostaining; original magnification (×200) shows adipoblasts with large, intensely stained nuclei (blue arrowheads). (C) Immunohistochemical image, P16 immunostaining; original magnification (×200) shows mature adipocytes of variable size (*) and spindle-shaped cells.

The patient underwent transoral endoscopic resection of the oesophageal lesion, using a transparent cap-fitted gastroscope to navigate the oesophagus. The mass was found to occupy approximately two-thirds of the circumference of the oesophagus, extending 38 cm from the left wall of the oesophageal inlet to the left posterior wall. Following haemostatic electrocoagulation of visibly engorged blood vessels using a disposable mucosal incision knife, the tumour was excised en bloc, beginning 1.5 cm distal to the site of coagulation. Although there was some intraoperative bleeding, it was managed effectively. The resected tumour measured approximately 22 cm in length and appeared largely intact (Figure 2A). A nasogastric tube was installed postoperatively. Histopathological examination of the specimen showed mucosal ulceration with underlying proliferation of spindle cells and blood vessels. The spindle cells were interspersed with mature adipocytes, and areas of myxoid degeneration were noted (Figure 2B and 2C). Immunohistochemistry revealed Vim(+), P16(+), MDM2 (weakly positive), CDK4(+), CD34(+), S100 (spindle cells negative, adipocytes positive), SOX10(-), Desmin(-), SMA(-), Caldesmon(-), Melan-A(-), HMB45(-), CD117(-), DOG-1(-), and Ki-67 (approximately 3% positivity). Fluorescence in situ hybridization (FISH) confirmed amplification of the MDM2 gene. The patient received supportive care postoperatively, including gastrointestinal decompression, anti-infective therapy, and symptomatic management. He was discharged one week later without complications.

## Discussion

Liposarcoma ranks among the most prevalent soft tissue sarcomas in adults,^1^ with peak incidence occurring between the ages of 40 and 60, and a slight male predominance.^2^ The tumours typically arise deep within soft tissues, such as in the extremities, the retroperitoneum, and the neck, while involvement of other anatomical sites is rare. Liposarcomas are classified histologically into four subtypes, namely, well-differentiated, myxoid, pleomorphic, and dedifferentiated,^3^^,^^4^ with the well-differentiated subtype being the most common. Although this subtype is locally aggressive, it generally lacks metastatic potential.^5^^,^^6^

Oesophageal liposarcomas are most often located in the cervical portion of the oesophagus, frequently presenting as large polypoid masses. These tumours can often exceed 10 cm in length, and, due to their size, may lead to mechanical obstruction. Documented tumour lengths range from 4 to 33 cm.^7^^,^^8^ Progressive dysphagia is the most common symptom, accompanied by weight loss, retrosternal pain, vomiting of the tumour into the mouth, and self-ingestion.^9^^,^^10^ Patients may also experience dyspnoea resulting from pressure exerted by the tumour on the respiratory tract.^11^ Imaging modalities, such as CT and MRI, may reveal fatty densities suggestive of liposarcoma. Well-differentiated liposarcoma shows distinctive fatty density (CT values ranging from −40 to −120 HU). On MRI, fatty tissue within the tumour exhibits signal similar to those of subcutaneous fat, with reduced signals in fat-suppressed sequences. Intralesional features include fine irregular septa, nodular non-adipose soft tissue components, and minute vascular shadows. Contrast-enhanced imaging reveals poor vascularization with minimal global enhancement, typically confined to the septa. MRI offers superior diagnostic value and assists in localization of the biopsy site.^12^Although GIST lesions may contain small amounts of fat, they more often present as hyper vascular masses. Polyps generally contain no fat. The pattern of enhancement is also important, with stromal tumours showing significant enhancement, while cord-like enhancement is seen in polyps, and liposarcomas show no significant enhancement. The enhancement pattern is also crucial for differentiation: GIST lesions show significant enhancement, polyps exhibit cord-like enhancement, and liposarcomas show no significant enhancement. Both upper gastrointestinal endoscopy and contrast-enhanced oesophagography provide valuable information about mucosal integrity, oesophageal peristalsis, and the extent of the lesion. Nonetheless, definitive diagnosis relies on histopathological and immunohistochemical evaluation.^13^

## Learning points

- Liposarcoma is a malignant tumour derived from adipocytes. The well-differentiated liposarcoma subtype, when occurring in the oesophagus, shows a relatively high degree of pathological differentiation. (characterized by tumour cell morphology being close to that of normal adipocytes) and tends to be a low-grade malignancy.
- Primary oesophageal liposarcoma is extremely rare and occurs mostly in the middle and lower segments of the oesophagus, which have relatively abundant adipose tissue. It is necessary to distinguish between primary liposarcoma tumours and those that have metastasized to the oesophagus from other sites.
- CT: CT shows fatty tissue density, with no enhancement of fat, but mild enhancement of the septa and solid components.
- The final diagnosis depends on pathology (biopsy/postoperative) and immunohistochemical and FISH detection of MDM2.

## Data Availability

Data will be made available on request.
